# Combined Tumors in Hematolymphoid Neoplasms: Case Series of Histiocytic and Langerhans Cell Sarcomas Arising From Low-Grade B-Cell Lymphoma

**DOI:** 10.1177/2632010X19878410

**Published:** 2019-09-29

**Authors:** Stephanie L Skala, Jing C Ye, Jennifer Stumph, William R Macon, Frances R Quinones, Vadim Khachaturov, Rhett P Ketterling, Rajan Dewar

**Affiliations:** 1 Department of Pathology, Michigan Medicine, University of Michigan, Ann Arbor, MI, USA; 2 Division of Hematology and Oncology, Department of Internal Medicine, University of Michigan, Ann Arbor, MI, USA; 3 Michigan Pathology Specialists, Spectrum Health, Grand Rapids, MI, USA; 4 Department of Laboratory Medicine and Pathology, Mayo Clinic College of Medicine, Rochester, MN, USA

**Keywords:** Transdifferentiation, histiocytic sarcoma, Langerhans cell sarcoma, chronic lymphocytic leukemia

## Abstract

We report an index case of histiocytic sarcoma arising in a 70-year-old patient with long-standing chronic lymphocytic leukemia/small lymphocytic lymphoma (CLL/SLL). The patient presented in 2017 with painful, enlarging swelling of the left neck. He had remote history of cutaneous squamous cell carcinoma with no sign of recurrence, and his CLL/SLL was thought to be in remission. Computed tomography showed mild splenomegaly and multifocal lymphadenopathy including a 3-cm left neck mass. Biopsy of the left neck mass showed CLL/SLL with associated histiocytic sarcoma. Flow cytometry demonstrated a B cell neoplasm with CLL/SLL phenotype. Despite radiation therapy, he expired 3 months after presentation. Two similar cases (CLL/SLL and histiocytic sarcoma, follicular lymphoma and Langerhans cell sarcoma) from another institution are also illustrated. The pathological features of combined tumors in lymphoid neoplasms, a general framework to the work-up to determine interrelatedness of tumor components, and the clinical relevance are discussed.

## Case 1

### Clinical history

A 70-year-old man was diagnosed with chronic lymphocytic leukemia (CLL) in 2009 when lymphocytosis was noted. Interphase fluorescence in situ hybridization (FISH) studies performed on peripheral blood at that time confirmed the presence of abnormalities including homozygous deletion of chromosome 13q. Flow cytometric evaluation of peripheral blood demonstrated positivity for ZAP70 in 13% of the CD19-positive B cells. His CLL was monitored until 2012 when patient had disease progression with development of diffuse lymphadenopathy and increasing splenomegaly and was treated with bendamustine and rituximab. He was then treated with ibrutinib in 2014. He presented in February 2017 to University of Michigan Hematology Clinic for a consult with a 2-month history of neck and face swelling, cough, dysphagia, and night sweats. The patient was admitted to a local hospital prior to this consult and underwent fine needle aspiration (FNA) of the left neck mass with pathology report showing persistent CLL. Given the rapid disease progression which was not consistent with CLL, the decision was made to get a positron emission tomography (PET)/computed tomography (CT) scan and excisional biopsy of the presumed cervical lymph node with the highest 18F-fluorodeoxyglucose (FDG) metabolic activity (standardized uptake value = 23). Computed tomography showed mild splenomegaly and multifocal lymphadenopathy including a 3-cm left neck mass.

### Histopathology

**Figure 1.**
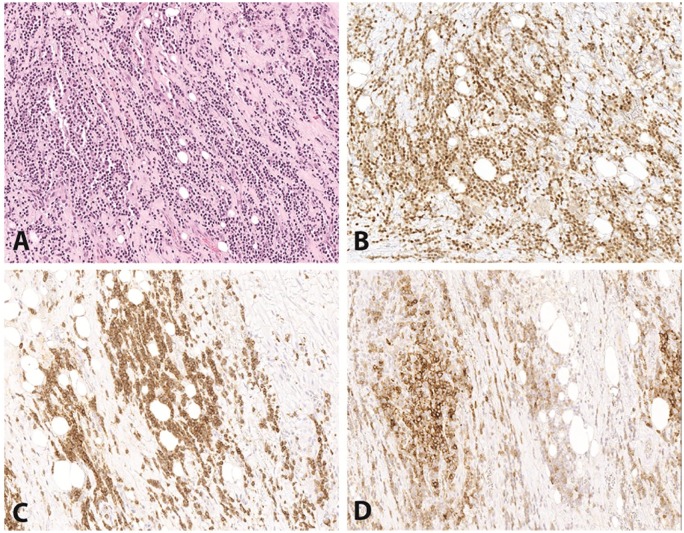
Case 1: chronic lymphocytic leukemia/small lymphocytic lymphoma (CLL/SLL). Some areas of this biopsy show a proliferation of monotonous small cells compatible with CLL/SLL (A, H&E, 200×). The small B cells are positive for PAX5 (B, 200×), CD5 (C, 200×), and CD23 (D, 200×). H&E indicates hematoxylin and eosin.

**Figure 2.**
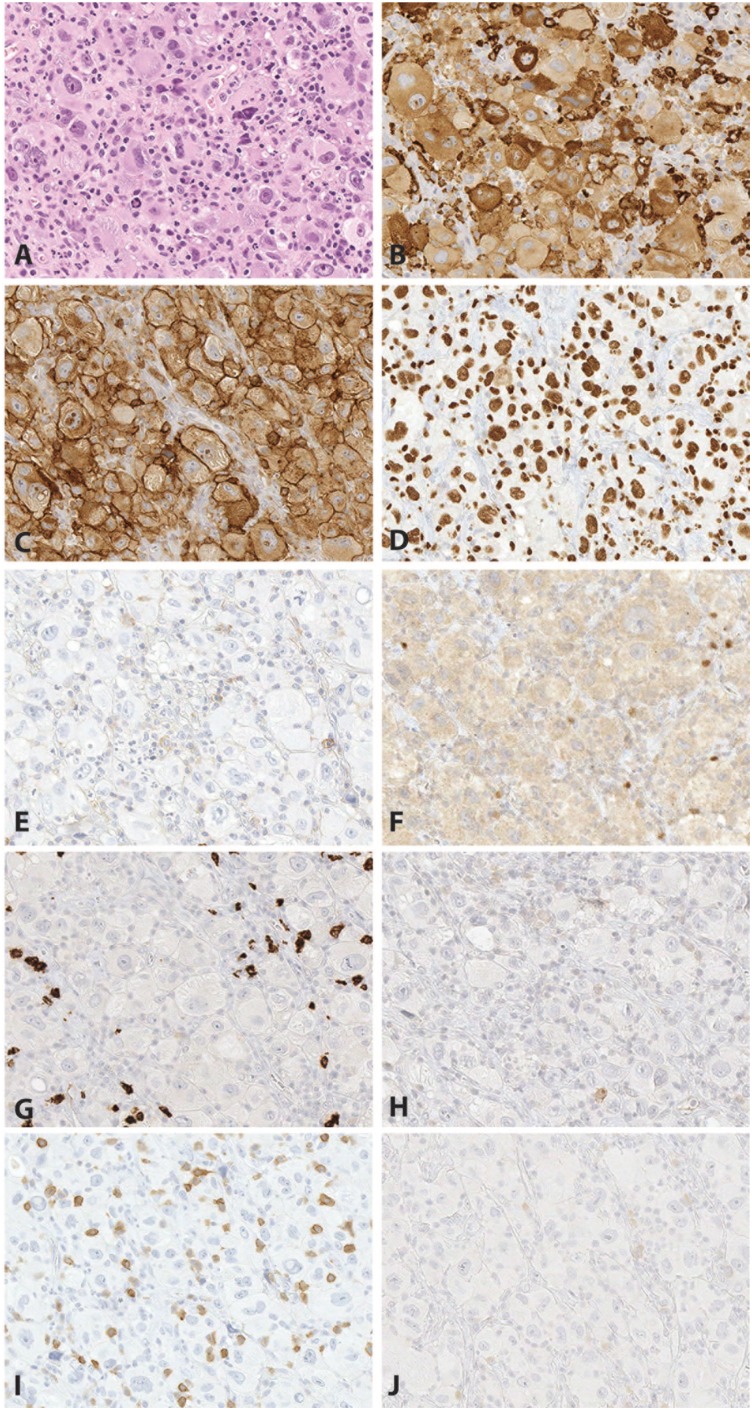
Case 1: atypical large cells arising in a background of chronic lymphocytic leukemia/small lymphocytic lymphoma (CLL/SLL). The large cells (A, H&E, 400×) are positive for CD163 (B, 400×), CD4 (C, 400×), and PU.1 (D, 400×), consistent with histiocytic differentiation. The large cells are negative for CD45 (E, 400×), PAX5 (F, 400×), CD15 (G, 400×), CD30 (H, 400×), CD5 (I, 400×), and CD23 (J, 400×). H&E indicates hematoxylin and eosin.

An excisional biopsy of a left neck level 2 mass was performed in February 2017. Microscopic images are shown in Figures 1 and 2. Sections showed soft tissue without definitive lymph node architecture infiltrated by small lymphoid cells with round nuclei, clumped chromatin, and scant to moderate cytoplasm. In addition to this, several foci with sheets of very large, unusually multinucleated cells with prominent nucleoli, occasional intracytoplasmic neutrophils and/or plasma cells, and increased mitotic activity are seen. These large cells are also seen scattered within the small lymphoid neoplasm. Scattered plasma cells and neutrophils were prominent.

### Immunohistochemistry

PAX5 highlighted numerous small B-cells with expression of CD5 and CD23 in a subset, consistent with a background of CLL/small lymphocytic lymphoma (SLL) (Figure 1). Although the large atypical cells had some morphological features suggestive of Hodgkin lymphoma, they were negative for CD15, CD30, PAX5, and CD45 (Figure 2). CD15 highlighted background granulocytes, and CD30 highlighted rare immunoblasts. The large atypical cells were negative for cytokeratin cocktail and EMA as well as HMB-45 and Melan-A, arguing against diagnosis of carcinoma or melanoma. ALK, CD21, CD23, CD79a, CD43, and EBER were all negative. Additional stains were added to evaluate for the possibility of histiocytic sarcoma. The large atypical cells were positive for CD68 (KP-1), CD163, S100, PU.1, CD4, CD14 (subset), and lysozyme, and negative for CD1a (Figure 2). The Ki-67 proliferative index was approximately 50% to 70% in the large cells, and approximately 10% overall.

### Flow cytometry

Flow cytometry performed on a core biopsy of the left neck mass demonstrated a kappa-restricted B cell neoplasm with expression of CD19 (dim), CD20 (dim), CD5 (dim), CD23 (dim-moderate), and FMC7 (dim, minor subset). This immunophenotype is consistent with CLL/SLL. A separate large cell component was not detected by flow cytometry.

### *Fluorescence* in situ *hybridization*

**Figure 3.**
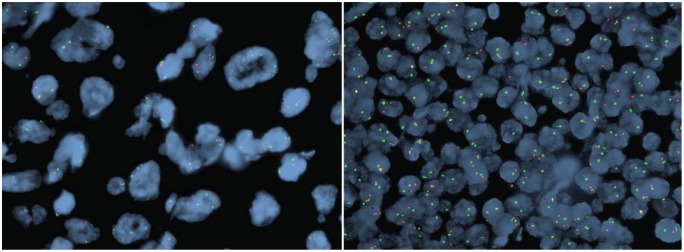
Case 1: clonally related chronic lymphocytic leukemia/small lymphocytic lymphoma (CLL/SLL) and histiocytic sarcoma. Fluorescence in situ hybridization confirms homozygous deletion of 13q14 in the large cells of histiocytic sarcoma (left) and the small cells of CLL/SLL (right). The probes shown here are D13S319 [13q14] (red) and LAMP1 [13q34] (green). Homozygous deletion is represented by 0 red signal and 2 green signals, and heterozygous deletion by 1 red signal and 2 green signals.

Fluorescence in situ hybridization studies were performed at Mayo Clinic College of Medicine (Figure 3) on formalin-fixed, paraffin-embedded tissue. The small cells (SLL) demonstrated a heterozygous or homozygous 13q deletion in approximately 90% of the nuclei. The very large cells (histiocytic sarcoma) demonstrated a complex, near-tetraploid result for all probes with relative loss of chromosome 13q in a pattern consistent with a “doubling” of a heterozygous 13q deletion and a homozygous 13q deletion. These FISH results suggest that the SLL and the histiocytic sarcoma are very likely related due to a complex chromosomal “doubling” of the SLL clone with retention of the heterozygous and homozygous 13q deletions. For reference, the normal cutoffs for paraffin-embedded tissue for this assay include <21% for 6q deletion, 11q deletion, 13q deletion, and 17p deletion; <10% for homozygous 13q deletion; <15% for trisomy 12; and <3.0% for *CCND1/IGH* fusion. Additional images of these results from our index case were presented in our recent review article,^1^ which sparked this multi-institutional case series.

### Follow-up

After the diagnosis of histiocytic sarcoma was made, the patient underwent radiation therapy due to symptomatic neck pain and dysphagia. Repeat bone marrow biopsy was not performed. Unfortunately, his performance status never improved to allow him to get further systemic treatment. He succumbed to disease 3 months after diagnosis.

## Case 2

**Figure 4.**
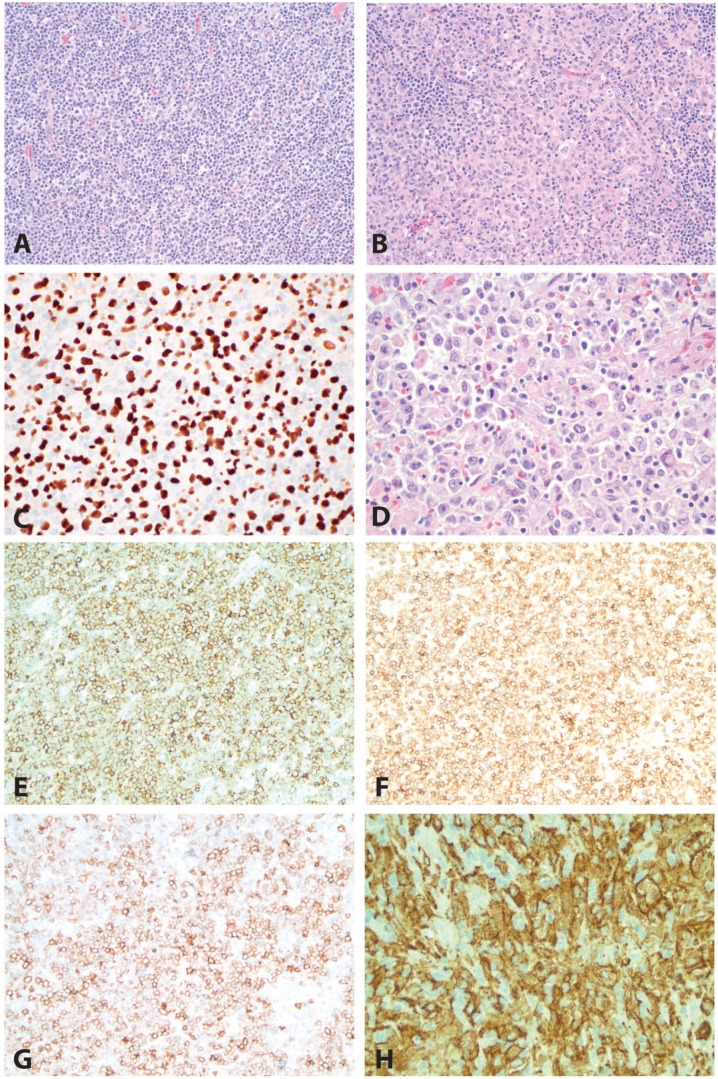
**C**ase 2: chronic lymphocytic leukemia/small lymphocytic lymphoma (CLL/SLL) and histiocytic sarcoma. This case showed CLL/SLL (A and B, H&E, 200×) in association with histiocytic sarcoma (B, H&E, 200× and D, H&E, 400×). A PU.1 immunohistochemical stain (C, 400×) demonstrated more intense expression in the histiocytic sarcoma than in the CLL/SLL. The CLL/SLL cells were positive for CD20 (E, 200×), CD5 (F, 200×), and CD23 (G, 200×). The histiocytic sarcoma cells were positive for CD163 (H, 400×). H&E indicates hematoxylin and eosin.

An 80-year-old woman presented with approximately 2 years of mildly progressive generalized lymphadenopathy and tremendous growth of a right axillary lymph node in last month. A 5-cm fleshy lymph node with areas of necrosis was submitted in formalin. Microscopic sections showed effacement of node architecture, with some areas showing a diffuse infiltrate of monomorphic small lymphocytes with clumpy chromatin, inconspicuous nucleoli, and scant cytoplasm. The small lymphoid cells were positive for CD19, CD20, CD45, CD79a, PAX5, CD5, CD23, CD43, and BCL2 and negative for CD3, CD10, CD21, and cyclin D1. Intermediate to large cells with oval/pleomorphic nuclei, dispersed chromatin, variably prominent nucleoli, and abundant pale eosinophilic cytoplasm were seen infiltrating between aggregates of small lymphoid cells. The large cells were positive for PU.1, CD4, CD43, CD45, CD68 (KP-1), CD163, and lysozyme. The final diagnosis was CLL/SLL and histiocytic sarcoma, possibly representing transdifferentiation of CLL to histiocytic sarcoma. The patient sought several treatment opinions but opted for comfort care and died approximately 3 months after diagnosis. Bone marrow biopsy and molecular testing were not performed for this case. This case is illustrated in Figure 4.

## Case 3

**Figure 5.**
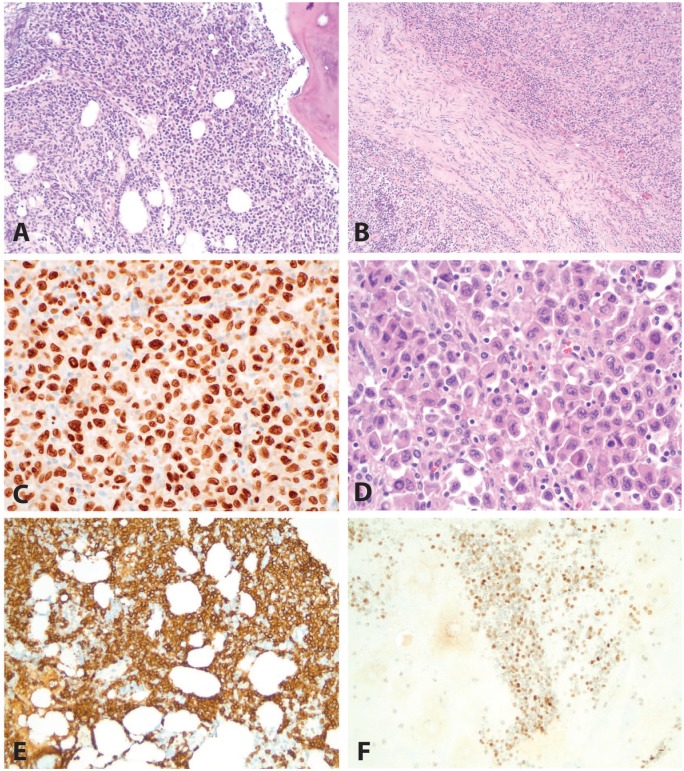
Case 3: low-grade follicular lymphoma and Langerhans cell sarcoma. This case shows a low-grade follicular lymphoma (A, H&E, 200× and B, H&E, 100×) in association with Langerhans cell sarcoma (B, H&E, 100× and D, H&E, 400×). A PU.1 immunohistochemical stain (C, 400×) demonstrates more intense expression in the Langerhans cell sarcoma than in the follicular lymphoma. The low-grade B-cell lymphoma was positive for CD20 (E, 200×) and BCL6 (F, 200×). H&E indicates hematoxylin and eosin.

A 65-year-old woman presented with abdominal pain and was found to have a mass in the manubrium. Core biopsy of the lesion showed trabecular bone infiltrated by a diffuse proliferation of small to medium-sized lymphoid cells with round to irregular nuclei, condensed chromatin, and indistinct nucleoli, as well as occasional small lymphoid cells with abundant pale cytoplasm. The small lymphoid cells were positive for CD20, BCL6 (weak, subset), and BCL2 and negative for CD3, CD5, cyclin D1, and CD23. Based on this biopsy, the patient received a diagnosis of low-grade B-cell lymphoma, with a differential including marginal zone lymphoma and follicular lymphoma. She returned 3 months later with shoulder pain and was found to have a pathological clavicle fracture. Core biopsy of this area showed Langerhans cell sarcoma (LCS), diffusely positive for CD1a and S100 with a Ki-67 proliferative index of 70%. Radical resection of the node, bone, and soft tissue was performed shortly thereafter, showing LCS with associated B-cell lymphoma. Immunoglobulin gene rearrangement studies demonstrated 2 prominent peaks of the same size in the LCS and the associated low-grade B-cell lymphoma, suggesting a clonal relationship between the 2. Foundation One testing (Foundation Medicine, Inc, Cambridge, MA, USA) performed on formalin-fixed, paraffin-embedded tissue from the resection including LCS and low-grade B-cell lymphoma identified *IGH-BCL2* rearrangement, *MAP2K1 (MEK1)* mutation (K59_V60insQK), *PTEN* loss, *CARD11* K215del, *CDKN2A*/*B* loss, *FAS* loss, *HISTH1D* S87fs*3, *SF3B1* E862K, *TNFAIP3* R685fs*3, and *TNFRSF14* loss. Microdissection was not performed to differentiate between the neoplasms; the sample included approximately 70% lymphoma and 30% sarcoma. A staging bone marrow biopsy demonstrated low-level involvement (less than 5%) by low-grade B-cell lymphoma (follicular lymphoma in light of the Foundation One results) and no evidence of LCS. Flow cytometric evaluation confirmed the presence of a small kappa-restricted B-cell lymphoma with dim CD10 expression, and no CD1a-positive events were identified. Positron emission tomography imaging showed widely metastatic disease in the lungs, lymph nodes, and bones. The patient underwent 2 cycles of chemotherapy with rituximab, ifosfamide, carboplatin, and etoposide (RICE) with partial response. She subsequently underwent radiation therapy with good response and is currently tolerating venetoclax well with no evidence of disease 10 months after diagnosis of LCS. This case is illustrated in Figure 5. Based on the Foundation One results, other potentially useful targeted therapies could include cobimetinib, trametinib, or binimetinib (due to the *MEK1* mutation) and temsirolimus, everolimus, or copanlisib (due to *PTEN* loss). There is a case report of a 62-year-old man with follicular lymphoma and histiocytic sarcoma with an activating *MAP2K1 (MEK1)* mutation, who showed complete clinical response and response on imaging to trametinib, an MEK1 and 2 inhibitor.^2^

## Discussion

Chronic lymphocytic leukemia/small lymphocytic lymphoma is fairly common, with an incidence of approximately 20 cases per 100,000 adults at age 70 years.^3^ It is a low-grade mature B cell neoplasm diagnosed in the peripheral blood when abnormal B cells with coexpression of CD5 and CD23 exceed 5000 cells per microliter and referred to as SLL when disease is predominantly based in the lymph nodes or spleen.^4^ It is characterized by small mature lymphoid cells with round nuclei and clumped chromatin. Pale pseudofollicles (“proliferation centers”) and scattered prolymphocytes with prominent nucleoli can be seen. Fluorescence in situ hybridization can be used to identify cytogenetic abnormalities including 11q del, trisomy 12, 13q del, and 17p del. Unfavorable outcomes (including progression with increased size of cells or proliferation centers and transformation to a higher-grade neoplasm such as diffuse large B cell lymphoma or classic Hodgkin lymphoma [cHL]) are associated with 11q del and 17p del, whereas isolated 13q del in a low number of cells is favorable.^3^ Patients with 13q deletion in >65.5% of the CLL nuclei have been shown to have a lower 5-year untreated rate than patients with isolated 13q deletion in a low number of cells.^5^ Our patient (case 1) had 13q deletion in most of his CLL cells and subsequently developed an associated histiocytic sarcoma with rapid clinical decline.

The hematology/hematopathology literature includes descriptions of “composite lymphomas” comprising 2 types of lymphoma which are sometimes clonally related and sometimes not.^4,6,7^ In more than half of composite classic cHL/non-Hodgkin lymphoma (NHL) as well as more than half of consecutive cHL and NHL, there is a clonal relationship suggesting that the lymphomas may share a common precursor.^7^ Mutation analysis often suggests a common origin rather than additional mutations leading to transformation. Importantly, low-grade lymphoma developing into high-grade lymphoma is referred to as transformation rather than composite lymphoma. In composite lymphoma composed of CLL and Hodgkin lymphoma, characteristic Reed-Sternberg cells and their typical milieu are seen in areas of the CLL.^7^ The Hodgkin variant of Richter transformation, on the contrary, is not considered to be a composite lymphoma.^8,9^

**Figure 6.**
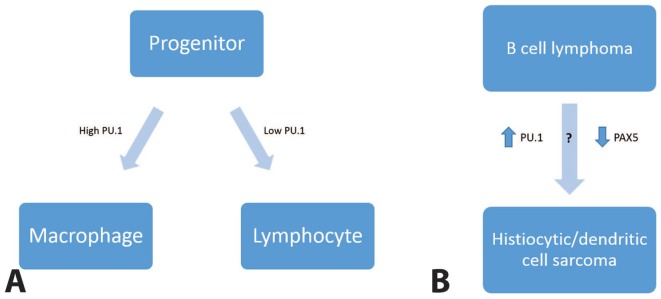
PU.1 and transdifferentiation. Upregulation of PU.1 is thought to allow lymphoid cells to switch to histiocytic differentiation.

Interrelated low-grade B cell lymphomas and histiocytic/dendritic cell sarcomas also exist. Although there is some evidence for a shared progenitor between B cell neoplasms and histiocytic/dendritic cell sarcomas (eg, clonal rearrangement of *IGH* and/or *IGK* genes in sporadic histiocytic/dendritic cell sarcomas),^10,11^ the predominant theory is that the sarcomas arise from low-grade B cell lymphoma through a process of “transdifferentiation,” or transformation of differentiated cells from 1 line of differentiation to another. The transcription factors PU.1 and C/EBP have been shown to be involved in transdifferentiation of B cells into macrophages in mouse models.^12,13^ PU.1 is needed for development of both macrophage and lymphoid lineages, and it appears to be the primary determinant between B cell lineage and macrophage lineage, with lower levels leading to B cell differentiation.^14^ Some histiocytic/dendritic cell sarcomas arising from follicular lymphoma have lacked PAX5 expression; attenuation of B cell transcription factors may also play a role in transdifferentiation.^15^ An illustration of this proposed mechanism is shown in Figure 6. All 3 cases described here demonstrated strong and diffuse PU.1 staining without significant expression of B cell markers in the histiocytic/dendritic cell sarcoma.

In 2009, Fraser et al^16^ published a case report describing a 77-year-old man with a 3-year history of CLL/SLL who had clinical evidence of disease progression and was found to have CLL/SLL in the bone marrow and a composite of CLL/SLL and interdigitating dendritic cell sarcoma in lymph nodes. Polymerase chain reaction (PCR) studies demonstrated identical IgH rearrangements in the 2 tumors, and the V-J junction sequences were confirmed to be identical. Other studies have also demonstrated identical IGH and/or IGK rearrangements in low-grade B cell lymphomas and associated secondary histiocytic sarcoma.^15,17,18^ Fraser et al compared mRNA expression of transcription factors in the CLL/SLL and interdigitating dendritic cell sarcoma described earlier. PU.1 mRNA and protein levels were increased 4-fold in the interdigitating dendritic cell sarcoma cells compared with the CLL cells, whereas CEBP alpha was decreased 5-fold in the interdigitating dendritic cell sarcoma cells.^16^ Both neoplasms demonstrated trisomy 12, with 16q gains in the interdigitating dendritic cell sarcoma by array comparative genomic hybridization. In a 2011 study of 7 cases of CLL/SLL with transformation to histiocytic/dendritic cell sarcomas, 2 had 17p deletion in both the CLL/SLL and sarcoma components.^19^ All cases of CLL/SLL with transformation to histiocytic/dendritic cell sarcoma expressed PU.1, and 1 expressed CD163 and was classified as histiocytic sarcoma.

Approximately 25% of the cases of histiocytic sarcoma result from presumed transdifferentiation from low-grade B cell lymphoma,^20^ and there are rare reports of LCS arising from low-grade B cell lymphoma.^19,21^ Both histiocytic sarcoma and LCS most commonly present as extranodal masses and are often associated with systemic (B) symptoms; however, the mass tends to be painless.^22^

Histiocytic sarcoma is composed of large (>20 µm) pleomorphic round to oval cells with abundant cytoplasm, ovoid to folded nuclear contours, vesicular chromatin, and distinct cell borders, often in a diffuse pattern.^23,24^ The neoplastic cells may have a xanthomatous appearance, form giant cells, or show hemophagocytosis or emperipolesis.^23^ CD163 expression is more specific to macrophages/histiocytes than CD68 and should show a membranous and cytoplasmic pattern.^19^ Histiocytic sarcoma arising from B cell lymphoma may continue to express B cell markers such as PAX5 (weak).^17,24,25^ Langerhans cell sarcoma also has an overtly malignant appearance, with only a minor subset of cells showing folded nuclei, nuclear grooves, and abundant cytoplasm typical of Langerhans cells.^24,26^ Small foci of eosinophils can be seen in the background, supporting the diagnosis. Langerhans cell sarcoma is positive for CD1a, S100, and CD207/langerin by immunohistochemistry. Chromosome 17p abnormalities have been reported to increase the risk of transdifferentiation to histiocytic or dendritic cell sarcoma.^11^ In Case 1, both the CLL/SLL cells and histiocytic sarcoma cells demonstrated del(13q) by FISH.

Due to the rarity of composite lymphomas and of transdifferentiation to rare histiocytic/dendritic cell sarcomas, the optimal treatment strategies are unclear. When different lymphomas develop sequentially, the literature suggests that they should be treated based on the disease diagnosed on biopsy.^27-29^ When Hodgkin lymphoma and an indolent B cell lymphoma present simultaneously, they can be treated with a regimen targeting Hodgkin lymphoma in addition to an anti-CD20 antibody.^30^ When there is an incidental finding of CLL in an asymptomatic patient without compromise of hemoglobin or platelet counts, there is no indication for CLL treatment. However, a specialist for the other tumor would be consulted for further evaluation and co-management. Unfortunately, it is unclear what the best treatment for histiocytic sarcoma might be. Although CHOP (cyclophosphamide, doxorubicin, vincristine, and prednisone) appears to be the most common regimen,^31^ neither neoadjuvant nor adjuvant therapy have been demonstrated to prolong survival.^20^ Even patients with localized disease fare poorly. Median survival is less than 3 years. Both of our patients with histiocytic sarcoma (Cases 1 and 2) did not live long after diagnosis, although radiation therapy was given for Case 1. Even less is known about treatment and prognosis of LCS arising in association with B cell lymphoma; however, our patient with this combination of findings (Case 3) is doing well on venetoclax with no evidence of disease 10 months after diagnosis.

## Conclusions

Low-grade B cell lymphomas such as CLL/SLL rarely transform to histiocytic sarcoma or other histiocytic/dendritic cell sarcoma. This is thought to be the result of “transdifferentiation” of the neoplastic cells from lymphoid to histiocytic differentiation. The morphological differential diagnosis for histiocytic sarcoma is broad and includes transformation of low-grade lymphoma to another high-grade hematological malignancy. In particular, CD163 is a helpful marker for histiocytic differentiation due to its increased specificity relative to CD68, which can also stain carcinoma and melanoma.
